# High-resolution atomic force microscopy as a tool for topographical mapping of surface budding

**DOI:** 10.3389/fcell.2022.975919

**Published:** 2022-10-12

**Authors:** C. Sbarigia, S. Tacconi, F. Mura, M. Rossi, S. Dinarelli, L. Dini

**Affiliations:** ^1^ Department of Biology and Biotechnology “C. Darwin”, University of Rome Sapienza, Rome, Italy; ^2^ Research Center for Nanotechnology for Engineering of Sapienza (CNIS), University of Rome Sapienza, Rome, Italy; ^3^ Department of Basic and Applied Sciences for Engineering, University of Rome Sapienza, Rome, Italy; ^4^ Institute for the Structure of Matter (ISM), National Research Council (CNR) Rome, Rome, Italy; ^5^ CNR Nanotec, Lecce, Italy

**Keywords:** extracellular vesicles, membrane buds, roughness, topographical mapping, high resolution microscopy

## Abstract

Extracellular vesicles (EVs) are membranous nanoparticles secreted by almost all cell types. Reflecting the physiopathological state of the parental cell, EVs circulate in all body fluids, reaching distant cell targets and delivering different bioactive cargoes. As biological carriers, EVs influence their microenvironment altering cellular responses, being considered promising biomarkers for both physiological and pathological conditions. EVs are heterogeneous in terms of size and composition, depending on cell type and exposure to stimuli, and different methods have been developed to characterize their morphological, biophysical, and biochemical features. Among them, electron microscopy (EM) is the main technique used, however, the lack of standardized protocols makes it difficult to characterize EVs with a good reproducibility, thus using multiple approaches may represent a way to obtain more precise information. Furthermore, the relationship between architecture and function, not only in a molecular, but also in a cellular level, is gaining growing emphasis, characterizing morphometric parameters may represent a distinct, but effective approach to study the physiopathological state of the cell. Atomic force microscopy (AFM), may represent a promising method to study in detail EVs dynamics throughout the cell surface and its variations related to the physiological state, overcoming the limits of EM, and providing more reliable information. In this study, human neuroblastoma SH-SY5Y cell line, a cellular model to investigate neurodegeneration and oxidative stress, has been used to perform a comparative morphological and quantitative analysis of membrane budding and isolated large vesicles-enriched (microvesicles-like vesicles; MVs) fraction from control or oxidative stressed cells. Our main goal was to build up a methodology to characterize EVs morphology and spatial distribution over the cell surface in different physiological conditions, and to evaluate the efficacy of AFM against conventional EM. Interestingly, both microscopy techniques were effective for this analysis, but AFM allowed to reveal a differential profiling of plasma membrane budding between the physiological and the stress condition, indicating a potential relationship between mechanical characteristics and functional role. The results obtained may provide interesting perspectives for the use of AFM to study EVs, validating a morphometric approach to understand the pathophysiological state of the cell related to EVs trafficking.

## Introduction

Extracellular Vesicles (EVs) are heterogeneous, nanosized membrane-enclosed particles released by prokaryotic and eukaryotic cells in both physiological and pathological conditions (Dini et al., 2020). Considering that EVs classification and nomenclature is not standardized, generally the term EVs include two main subtypes, microvesicles (MVs) and exosomes (EXOs), also referred to as large and small vesicles. MVs are released directly from the plasma membrane, with a size range between 150 nm and 1 µm in diameter, while EXOs originate from the endocytic pathway, with a diameter between 40 and 160 nm (Kalluri and LeBleu, 2020). Once released in the extracellular milieu, EVs circulate in all body fluids and are carriers of different bioactive cargoes, e.g., proteins, lipids, metabolites, and nucleic acids, to distant or nearby target cells (Mckelvey et al., 2015; Van Niel et al., 2018). For this reason, EVs are considered important regulators of intercellular communication, involved in several physiological and pathological processes (Panfoli et al., 2018; He et al., 2021). Generally, to study EVs the first step required is the isolation, which can be performed by several methods that can be used also in combination (Böing et al., 2014; Tzaridis et al., 2021). Once isolated, it is pivotal to properly characterize EVs in their morphological, biophysical, and biochemical features. However, due to their heterogeneity in size and composition and the lack of standardized protocols, it is hard to quantify and characterize EVs with a good reproducibility (Božič et al., 2021). Different techniques have been developed to study EVs in terms of size, distribution, topology, and phenotype. Nevertheless, the most widely used technique for EVs imaging is Electron Microscopy (EM), with a resolution of 1–3 nm for Transmission Electron Microscopy (TEM) and 5 nm for Scanning Electron Microscopy (SEM). Even if a low-throughput analysis, since allowing to observe only several particles at a time, EM gives detailed information about the size, shape, and morphology of the sample. Next to EM, stands out Atomic Force Microscopy (AFM) that, by scanning the sample using a micrometric cantilever with a nanosized tip, provides, with a single exposure, not only a high-resolution imaging, but also important information about biophysical and biomechanical properties of the specimens, such as local elasticity or the surface roughness (Dinarelli et al., 2018a; Malenica et al., 2021). EVs have been investigated also at functional level, e.g., high-throughput OMICS techniques that allow to study EVs cargo, and the existence of a relationship between architecture and function, not only at a molecular, but also at a cellular level, is gaining growing importance. In this context, characterizing morphometric parameters may represent a distinct, but effective approach to study the pathophysiological state of the cell. Several studies have been exploited AFM to perform analysis at a nanoscale level of plasma membrane mechanical properties in response to biochemical stimuli (Girasole et al., 2012; Dinarelli et al., 2018b; Şahin et al., 2021). However, to the best of our knowledge, the use of morphometric parameters to describe EVs trafficking and its variations under certain physiological conditions, has not yet been investigated. EVs biogenesis and release has been proven to be increased under several stress conditions, including oxidative stress (Qi et al., 2021). Resulting from the accumulation of free radicals, oxidative stress alters biological function of macromolecules, such as proteins, lipids, and nucleic acids, determining harmful downstream effects. Furthermore, oxidative stress has been proven to modulate the level and content of released EVs, depending on the cell type and stimulus (Chiaradia et al., 2021). Several studies have demonstrated that an increase of EVs release in oxidative stress conditions may occur, driven by different factors causing membrane rearrangements, fluidity alterations, and cytoskeleton remodeling (Atienzar-Aroca et al., 2016; Holliday et al., 2019; Record et al., 2018; Van Meteren et al., 2019; Wilson and González-Billault, 2015). For this reason, EVs are implicated in diseases strictly linked to oxidative stress, for instance neurodegenerative disorders (Fowler and Hill, 2019; Meldolesi, 2021; Qi et al., 2021). Human neuroblastoma SH-SY5Y cell line, widely used as a model to investigate neurodegeneration and oxidative stress (Martínez et al., 2020; Greco et al., 2021; Chen et al., 2022), has been chosen to perform a comparative morphological and quantitative analysis of membrane budding and isolated microvesicles-like vesicles (MVs), in physiological conditions (control) or under oxidative stress (H2O2-induced), by electron microscopy (EM) and atomic force microscopy (AFM). The main goal of this work was to define a new method to study EVs morphological features and their alterations under stress conditions, evaluating the efficacy of the system compared to conventional methods. Both the microscopy techniques were used to obtain the average values of vesicles size as well as their distribution, resulting increased after H2O2 treatment. However, AFM analysis allowed to obtain a more accurate topography and distribution of plasma membrane budding throughout the cell surface and to appreciate details and features not accessible from the EM. We performed the mapping of surface budding along two specific selected areas (the nuclear zone and the interposed area between perinuclear region and cellular edge) of control and oxidative-stressed cells, and used a morphometric parameter, the surface roughness, to get a relationship between the topography and the entity of the budding phenomenon, according to different physiological conditions. Taken together, our results may provide interesting perspectives to implement a morphometric approach for studying and understanding the pathophysiological state of the cell related to EVs trafficking.

## Materials and methods

### Cell culture and treatments

Human neuroblastoma SH-SY5Y cells were cultured in DMEM 4.5 g/L glucose (Sigma Aldrich, St. Louis, MO) supplemented with 10% heat-inactivated Fetal Bovine Serum (FBS) (Sigma Aldrich, St. Louis, MO), 2 mM l-glutamine (Corning, Manassas, VA), 100 UI/mL penicillin and streptomycin solution (Corning, Manassas, VA) and 10.000 U/mL amphotericin B (antimycotic solution) (Sigma Aldrich, St. Louis, MO) in a 5% CO2 humidified atmosphere, at 37°C. Cells were maintained in 75 cm^2^ flasks at a concentration of 2 × 106 cells/mL by passage every two to three days. To induce oxidative stress, cells were treated with H2O2 (Carlo Erba, Milan, Italy) at a final concentration of 100 μM for 1 h, followed by 24 h of recovery in EVs-depleted medium. After performing the treatments, cells were harvested for further analyses.

### Microvesicles-enriched fraction isolation by ultracentrifugation

Before the treatments, complete DMEM growth medium was overnight centrifuged at 110,000 *g* in order to remove contaminant FBS-derived EVs. After the treatments, MV-enriched fractions were concentrated from H2O2-treated and control SH-SY5Y cells conditioned culture medium by differential centrifugation, as described in (Panzarini et al., 2020). Briefly, the culture medium of each sample was first centrifuged at 500 g for 10 min at room temperature, and the resulting supernatant was then centrifuged at 800 g for 10 min at room temperature. The last centrifugation of the supernatant was performed at 2000 g for 20 min at room temperature in order to remove dead cells or cell aggregates. Cell-depleted supernatant was then centrifuged at 20.000 g for 20 min at 4°C using a Beckman Coulter Ultracentrifuge Optima XE (Beckman Coulter, Brea, CA), and the resulting MV-enriched pellet was collected and used for further analysis (i.e., the microvesicles-enriched fraction; MVs).

### Scanning and Transmission Electron Microscopy

H2O2-treated and untreated SH-SY5Y cells cultured on glass coverslips were fixed with 2.5% glutaraldehyde in 0.1 mol/L cacodylate buffer (pH 7.4) for 1 h at ice temperature and postfixed with 1% OsO_4_ in the same buffer for 1 h on melting ice. After fixation, cells were dehydrated with ethanol (EtOH), 50%, 70%, 95%, and 100%, followed by chemical drying with EtOH and Hexamethyldisilazane (HMDS) (Sigma-Aldrich, St. Louis, United States), 2:1, 1:1, 1:2, and finally in pure HMDS, evaporated overnight. Consequently, the specimens were mounted on aluminum stubs and 3 nm chromium-coated with a Quorum Q150 T sputter (Quorum Technologies, United Kingdom) in order to increase the electron conductivity. For TEM analysis, cells were fixed with 2.5% glutaraldehyde in 0.1 mol/L cacodylate buffer (pH 7,4) for one hour at ice temperature and post-fixed with 1% OsO4 in the same buffer for two hours at ice temperature. After fixation, cells were dehydrated with ethanol (25%, 50%, 70%, 90%, and 100%) and embedded in Spurr resin. Thin sections of 60 nm were cut at the ultramicrotome (Powertome PC, RMC Boeckeler Instruments, Germany) and deposited on 200 mesh copper-grids. After isolation, MVs from all experimental groups were fixed with 0.1% paraformaldehyde (PFA) in DPBS for 30 min at room temperature. Fixed samples were stained with 2% uranyl acetate for 7 min at room temperature, loaded on 200 mesh carbon-coated grids for the TEM observation. SEM and TEM analysis were both performed using a Zeiss Auriga Scanning Electron Microscope (Zeiss, Oberkochen, Germany) equipped with the STEM module, operating at 8 and 20 keV, respectively.

### Atomic force microscopy preparation procedure and imaging of cells and isolated microvesicles

For Atomic Force Microscopy, control and H2O2-treated human neuroblastoma SH-SY5Y cells were seeded in 6-well multiwell with coverslips at a concentration of 8 × 104 cells/cm2, and incubated overnight in a 5% CO2 humidified atmosphere, at 37°C. Cells were then washed twice in DPBS and fixed in 1% glutaraldehyde in 0.1 mol/L cacodylate buffer pH 7.4 at ice temperature for 30 min. After fixation, cells were washed again twice in DPBS and two final washes in bidistilled water were performed. The isolated microvesicles-enriched fractions were fixed with 0.1% paraformaldehyde (PFA) in DPBS for 30 min at room temperature and diluted in water. The AFM images were acquired in contact mode with a Multimode AFM (Bruker, Santa Barbara, CA). We used commercially available AFM tips, model DNP (Bruker, Santa Barbara, CA) with triangular shaped cantilever, nominal elastic constant of 0.06 N/m and nominal tip radius of 10 nm. The interaction force between the tip and the sample was selected to be less than one nN, in such a way to not damage both tip and sample. To gain an overview of the sample morphology two different strategies were adopted: when imaging cells the scan area was set to 60 microns and a subsequent zoom of 20 micron has been performed on an area of interest; while, when imaging microvesicles, a few images of 20 micron were acquired and a greater number on images of 10 micron were taken until a sufficient number of vesicles were measured. According to the selected scan range the speed was set to 1–4 s per line while at least 512 points per line were used in all acquired images.

### Atomic force microscopy images analysis and vesicles counting

The analysis of the AFM images was performed with the freely available software Gwyddion (www.gwyddion.net). All images were minimally treated (i.e., a mean plane subtraction, substrate tilt correction and line coupling) in order to avoid the thermal drift and the tilting of the sample. The isolated vesicles were counted by using Gwyddion’s routine “Mark Grains by Segmentations,” changing the working parameters according to the best possible identification of the vesicles. This very same approach can’t be used to directly measure the budding on the cellular membranes since their big and inhomogeneous variation in height (i.e., thicker and round shaped in the nuclear region, flat on the border) makes impossible to find a set of parameters that allow a good identification of the vesicles. Two different options were then available: 1) to deeply modify the images or 2) to select a different counting method (i.e., by hands), we preferred the latter since the former can induce systematic and unpredictable errors.

### Roughness measurements

AFM surface root mean square deviation (roughness; Rq) analysis of cell surface was performed by ImageJ Software version 1.8.0. Square 6 × 6 μm^2^ regions were randomly selected, and five measurements were taken from each sample. Representative 3D reconstructions of AFM images were also performed with the same software.

### Statistical analysis

Results are expressed as means ± SEM. Multiple comparisons were performed by two-way ANOVA. Comparisons between two groups were performed using a student’s t-test. Comparisons between two distributions were analyzed using a Kolmogorov-Smirnov test (GraphPad Prism 9 software, GraphPad Software, San Diego, CA**).**

## Results

### Morphological analysis of membrane budding in control and H202-treated SH-SY5Y cells by scanning electron microscopy and atomic force microscopy

**FIGURE 1:**
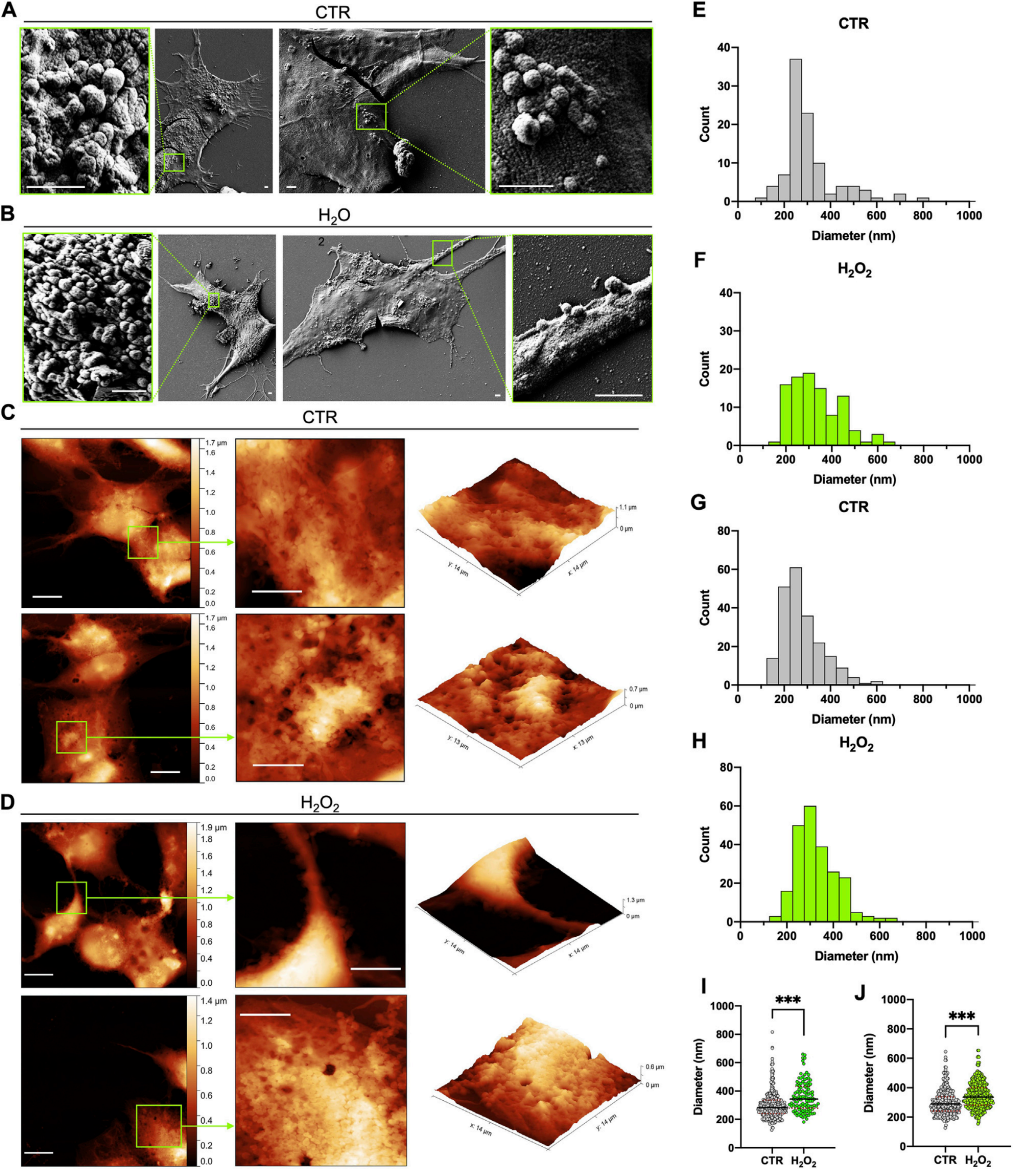
Comparison between SEM and AFM imaging of dried cells. **(A)** and **(B)** 2 typical SEM images acquired on the control and H2O2 treated cells, respectively, that show the presence of buds on the surface of the cells in localized regions; for each image a zoom on one of these regions is highlighted in a green box (scale bar 1 µm). **(C)** and **(D)** 2 typical AFM images acquired on the control and H2O2 treated cells, respectively, that show the presence of extensive budding on the surface of the cells; for each image (left side, scale bar 10 µm) a zoom on a region of interest highlighted in green is reported in 2-D (centre, scale bar 6 µm) and 3-D (right) views in order to better visualize the spatial arrangement of the membrane. **(E)** to **(H)** statistical analysis performed on the size distribution of the vesicles measured from all the SEM **(E,F)** and AFM **(G,H)** images acquired on the control **(E,G)** and H2O2 **(F,H)** treated cells. **(I)** and **(J)** dot plots of the direct comparison between the particle distribution of buds obtained on control (grey) versus H2O2 treated (green) cells from SEM [**(I)**, median diameter of 284 ± 5 nm for the control cells and 343 ± 10 nm for the H2O2-treated ones] and AFM [**(J)**, median diameter of 289 ± 4 nm for the control cells and 337 ± 4 nm for the H2O2 treated ones] images. For both techniques the obtained distributions are statistically different (*p* < 0.05).

An AFM and SEM microscopy analysis of vesicle budding at the cell surface of SH-SY5Y cells with and without H2O2 treatment were performed and the results are shown in Figure 1. SEM micrographs acquired on control and H2O2-treated SH-SY5Y cells showed cellular surfaces with numerous bulges and protruding buds of various sizes (Figures 1A,B). As a result of a qualitative analysis of the SEM images (Figures 1A,B), no differences were observed between the control and H2O2-treated groups in terms of budding entities and vesicles distribution. However, the histograms of Figures 1E,F show significant differences in the size distribution of buds between the control and H2O2 groups. Although the measured diameters range is similar (150–550 nm for control and 200–600 nm for H2O2-treated cells), the budding vesicles in SH-SY5Y cells exposed to H2O2 (Figure 1F) appear as a more homogeneous population with a comparable number of vesicles at different diameters with respect to control cells (Figure 1E) that resemble a very narrow distribution. On the other hand, the AFM microscopy analysis clearly shows that both control and H2O2-treated cells have a very irregular cell surface, characterized by an extensive budding of vesicles, large projections, and bulges (Figures 1C,D). In the case of control cells, these vesicular formations are primarily confined to the central part of cell bodies (Figure 1C), while in those treated with H2O2, the budding is pronounced even in the terminal portions, such as the cell periphery, protrusions, and cell processes (Figure 1D), something that was not observed in the SEM images. The histograms obtained from the sizing performed by AFM are shown in Figures 1G,H, the buds of both controls and H2O2-treated cells have a similar distribution but with a significant shift toward higher diameter values in the H2O2-treated cells. A direct comparison between the particle distribution of buds obtained, is represented in the dot plot graphs shown in Figures 1I,J where, sizing samples both with SEM (Figure 1I) and AFM (Figure 1J), shows a very good agreement between the two techniques and demonstrate a significant difference between control and H202-treated cells. Moreover, the median diameter analyzed from the SEM measurements of the control cells was 284 ± 5 nm, while for the H2O2-treated cells 343 ± 10 nm (Figure 1I). Both these values are well comparable with the AFM measured ones: 289 ± 4 nm for the control cells and 337 ± 4 nm for the treated ones (Figure 1J). Since the values obtained from both SEM and AFM analysis are similar, these results gave us a first insight of the potency of AFM technique as a valid system to characterize extracellular vesicles, as well as electron microscopy.

### Comparative morphological and quantitative analysis of isolated microvesicle-enriched fraction by Transmission Electron Microscopy and atomic force microscopy

**FIGURE 2:**
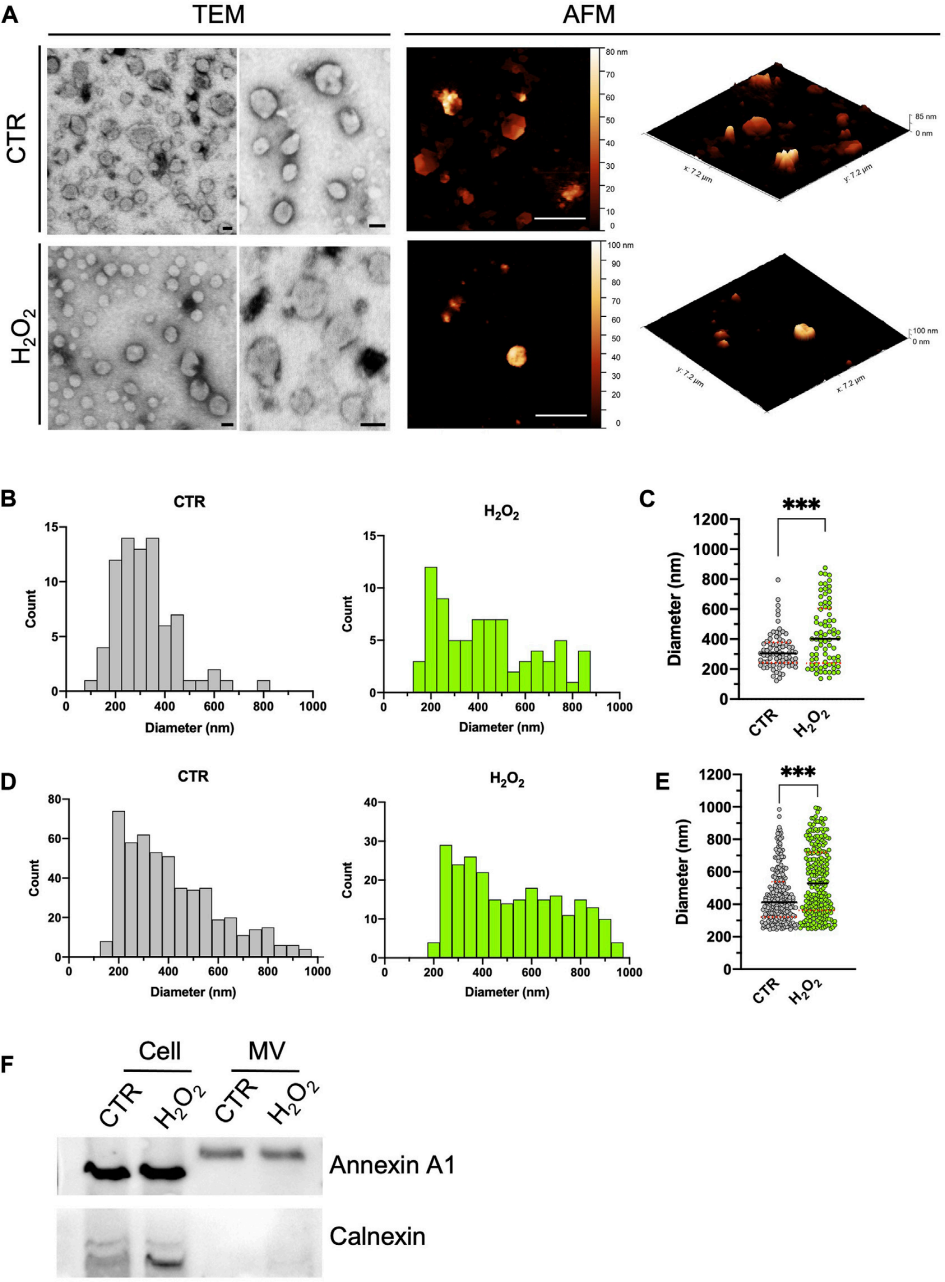
Comparison between STEM and AFM imaging on isolated vesicles. **(A)**: two typical STEM (left, scale bar 100 nm) and AFM (right, scale bar 2 µm) images acquired on isolated budding vesicles derived from control (first row) and H2O2 treated cells (second row). For each AFM image is also shown in its native 3D-view to better highlight the shape of the vesicles. **(B)** to **(E)**: statistical analysis of the size distribution of the vesicles as measured with the STEM **(B,C)** and AFM **(D,E)** performed of vesicles derived from control (grey values) and H2O2 treated cells (green values). A direct comparison between the two size distributions is shown for STEM [**(C)**, median diameter of 305 ± 14 nm for the vesicles derived from control cells and 401 ± 24 nm for the ones derived from H2O2-treated cells] and AFM [**(E)**, median diameter of 412 ± 10 nm for the vesicles derived from control cells and 528 ± 13 nm for the ones derived from H2O2-treated cells] data. For both techniques the obtained distributions are statistically different (*p* < 0.05). **(F)**: SDS-PAGE gel electrophoresis showing the presence of Annexin A1 (microvesicles marker) and calnexin (endoplasmic reticulum marker).

After concentration by ultracentrifugation, the morphology, size distribution, and quantity of isolated microvesicles were analyzed by TEM and AFM in the MV-enriched fraction obtained from control and H2O2-treated SH-SY5Y cells (results are summarized in Figure 2). As shown in Figure 2A, TEM micrographs of enriched fractions (left-sided) obtained from H2O2-treated and control cells revealed a heterogeneous population of rounded shape vesicles with a characteristic range diameter compatible with large vesicles, such as MV. Furthermore, the presence of the MV-associated marker annexin A1 (Jeppesen et al., 2019) and the absence of calnexin, generally used as a negative control, demonstrate the effective presence of MV in the isolated fractions and the quality of the isolation method (Figure 1F). The same landscape arises from the AFM images, shown on the right side of Figure 2A, where the vesicles’ morphological appearance and range diameter are essentially the same. Moreover, it is also possible to visualize the real 3D structure of the vesicles by taking advantage of the 3D metrological capabilities of the AFM. In Figure 2B, the histogram of the MV measured with TEM is reported. Control MV (gray graph) showed a diameter ranging between 123 and 794 nm with a single peak distribution. Conversely, the MV-enriched fraction from H2O2-treated cells (green graph) contains particles with a diameter range of 137–874 nm, wider than the MV-derived from control cells. In Figure 2C, the histograms of the size distribution obtained from the AFM images is reported. The diameter range of MV obtained from control SH-SY5Y cells (gray graph) is in the range 150–800 nm, lower than the measured values for vesicles from H2O2-treated cells (green graph) that ranges from 200 to 950 nm. The general qualitative behavior and appearance of the obtained distributions is consistent among the two techniques. More in detail, the distributions obtained between control and H2O2 differ in shape (i.e., in H2O2-treated cells, MV displays a more heterogeneous population of vesicles with a more variable diameter), thus indicating a high degree of comparability between the two methods. When comparing the mean values obtained for the two distributions, some differences arose, as indicated by the dot plot shown in Figure 2C for the TEM data and Figure 2E for the AFM data. The TEM states a median size of 305 ± 14 nm for the control and 401 ± 24 nm for the H2O2 treated, while the AFM gave a median diameter of 412 ± 10 nm for the control and 528 ± 13 nm for the H2O2 treated. The values measured by the AFM are significantly higher with respect to the ones obtained by the TEM. However, the distribution of values is perfectly compatible among AFM and TEM data (Figures 2C versus 2E), with the AFM presenting a definitely higher statistic. This is the major advantage of the AFM technique over the TEM, the number of particles that can be counted (by using the same acquisition time) is higher in the AFM case (for example, in our experiments for the control sample a total of around 90 MVs were counted from the TEM images, while for the AFM around 500 MVs) and this is crucial to obtain more meaningful and statistically consistent results.

### Mapping of surface budding in the nuclear and perinuclear/cellular edge area by atomic force microscopy

**FIGURE 3:**
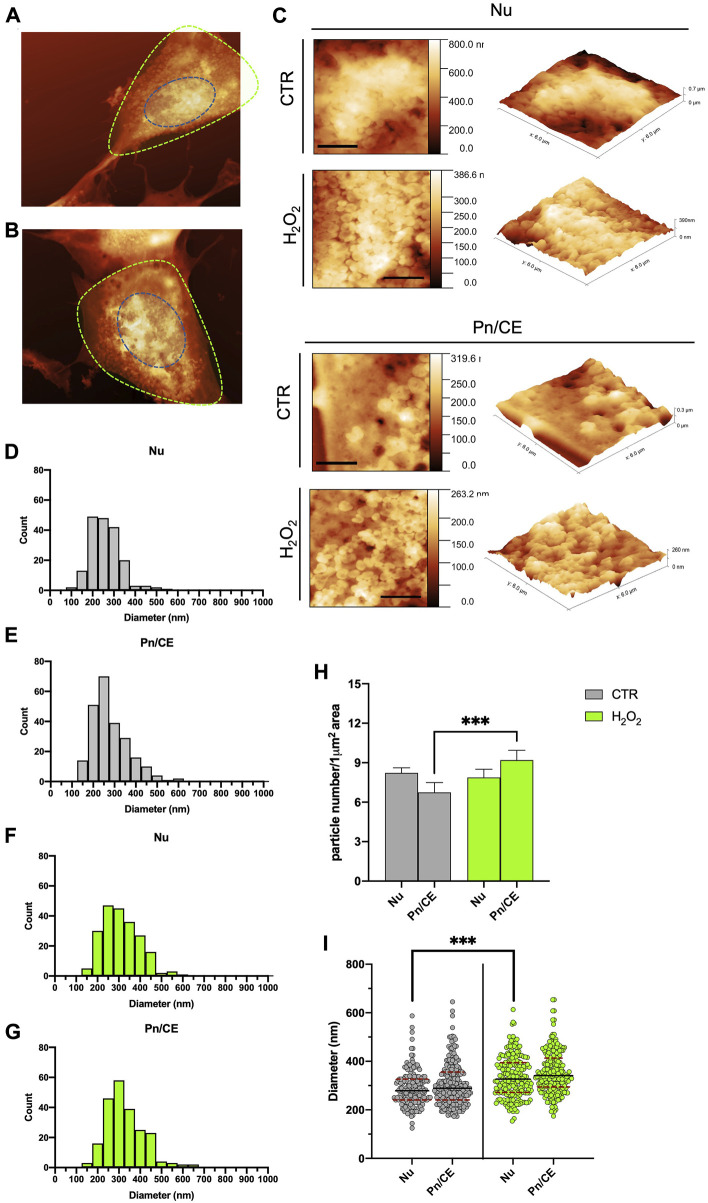
Statistical analysis of the spatial distribution of the budding vesicles on the cells, as seen with AFM. **(A)** and **(B)** example of the segmentation of the AFM images in nuclear (Nu, blue dotted line) and perinuclear (Pn/CE, green dotted line) for one control **(A)** and one H2O2 treated **(B)** cell. **(C)** typical high resolution 2D (left, scale bars 2 µm) and 3D (right) zoom of a 6 × 6 micron areas of the control and H2O2 treated cells in the Nuclear (top panels) and perinuclear (bottom panels) used for the manual counting and diameter measurements of the vesicles. **(D)** to **(G)** size distributions of the vesicles separated according to the sampling region [nuclear **(E,F)**, perinuclear **(E,G)** and the treatment (control cells showed in grey and H2O2 cells showed in green]. **(H)** comparison among the obtained data in terms of density of vesicles (i.e., number of vesicles measured for 1 µm^2^), the results obtained in the perinuclear areas of control and H202 treated cells are statistically different (*p* < 0.05). **(I)** direct comparison between the diameter distributions obtained for control (grey dots) and H202 treated (green dots) cells. The average diameter value obtained for the control cells are 279 ± 5 nm and 289 ± 6 nm in the nuclear and perinuclear regions, respectively, while for the H2O2 treated ones the corresponding values are 327 ± 6 nm and 341 ± 6 nm. The average diameters values in the nuclear regions were statistically different (*p* < 0.05) between control and H2O2 treated cells.

Microvesicles are released from cell membranes at the levels of specific areas, where lipid rafts and the molecular machinery involved in membrane curvature, vesicles detachment and cargo sorting are concentrated (Pollet et al., 2018; Elsherbini et al., 2021). As a consequence, this process can preferentially localize in certain parts of the cell in response to specific treatments or stimuli (Cloos et al., 2020; Chiaradia et al., 2021). By analyzing the AFM images, we mapped the surface budding on two specific areas of control and H2O2-treated cells, in order to determine which cell area was responsible for an increased release of vesicles. More in detail, we splitted the cells in two regions according to the measured height: 1) the higher part of the cell as the nuclear zone (Nu; blue dotted line in Figures 3A,B) and 2) the residual part as an interposed area between the perinuclear region and the cellular edge (Pn/CE; green dotted line in Figures 3A,B). Representative AFM magnifications of the nuclear area are reported in Figure 3C (upper panel). These images show a perfect example of how there are no significant variations in surface budding between the control and H2O2-treated cells in the nuclear area. On the contrary, in the lower panel of Figure 3C, are reported two explicative images of the increase in the budding process that can be observed in the Pn/CE area after treatment with H2O2. The same images are also shown in 3D-view (Figure 3C, right panels), in order to further highlight the presence of extensive surface blebbing in the nuclear regions of both samples, while the blebbing in the Pn/CE zone results clearly stronger in H2O2-treated cells when compared to the control group. We decided to quantify this variation among samples and regions by counting budding vesicles in defined square areas of 6 × 6 μm2. The results obtained for the Control cells are shown in Figures 3D,E for the Nuclear and Perinuclear areas, respectively, while for the H2O2 treated cells the results are shown in Figures 3F,G. Since the obtained particle size distributions are very similar to one another, to highlight subtle differences we decided to compare the data performing a normalization procedure, i.e., the results were expressed as the number of particles for μm^2^, and the obtained graph is shown in Figure 3H. From this comparison, it is possible to state that the treatment with H2O2 resulted in a statistically significant increase (*p* < 0.001) in buds density in the Pn/CE area, while this did not happen in the nuclear area. In addition, we analyzed the size distribution of budding vesicles in the Nu and Pn/CE areas of control and H2O2-exposed cells, to determine if the treatment affected not only the number of buds, but also their size, and the obtained graph is reported in Figure 3I. When considering the nuclear region, the H2O2 exposure did not modify considerably the size range of budding vesicles (125–587 nm for control vs. 154–613 nm for treated cells), but their distribution and median diameter (279 ± 5 nm for control vs. 327 ± 6 for H2O2-treated cells) changed significantly (*p* < 0.001). On the other hand, no significative differences in terms of range diameter and distribution of buds were found in the Pn/CE zone (173–645 nm; Figure 3E) when compared to the control (174–654 nm; Figure 3G); while an increase in the median diameter of buds was observed in the Pn/CE area of H202-treated SH-SY5Y cells (341 ± 6 nm) with respect to the control cells (289 ± 6 nm) (Figure 3I). Considering these observations, in control cells the vesicles budding appears homogeneous all over the cellular membrane, while the exposure to H2O2 exerts two main effects: 1) increasing the vesicles average size both in the nuclear and in the perinuclear regions, and 2) increasing the number of vesicles produced only in the perinuclear area. These considerations further confirm the unique capabilities of the AFM to give local information all over the cellular membrane, in response to a particular stimulus, specifically H2O2 treatment.

### Roughness measurement in the nuclear and perinuclear/cellular edge area

**FIGURE 4:**
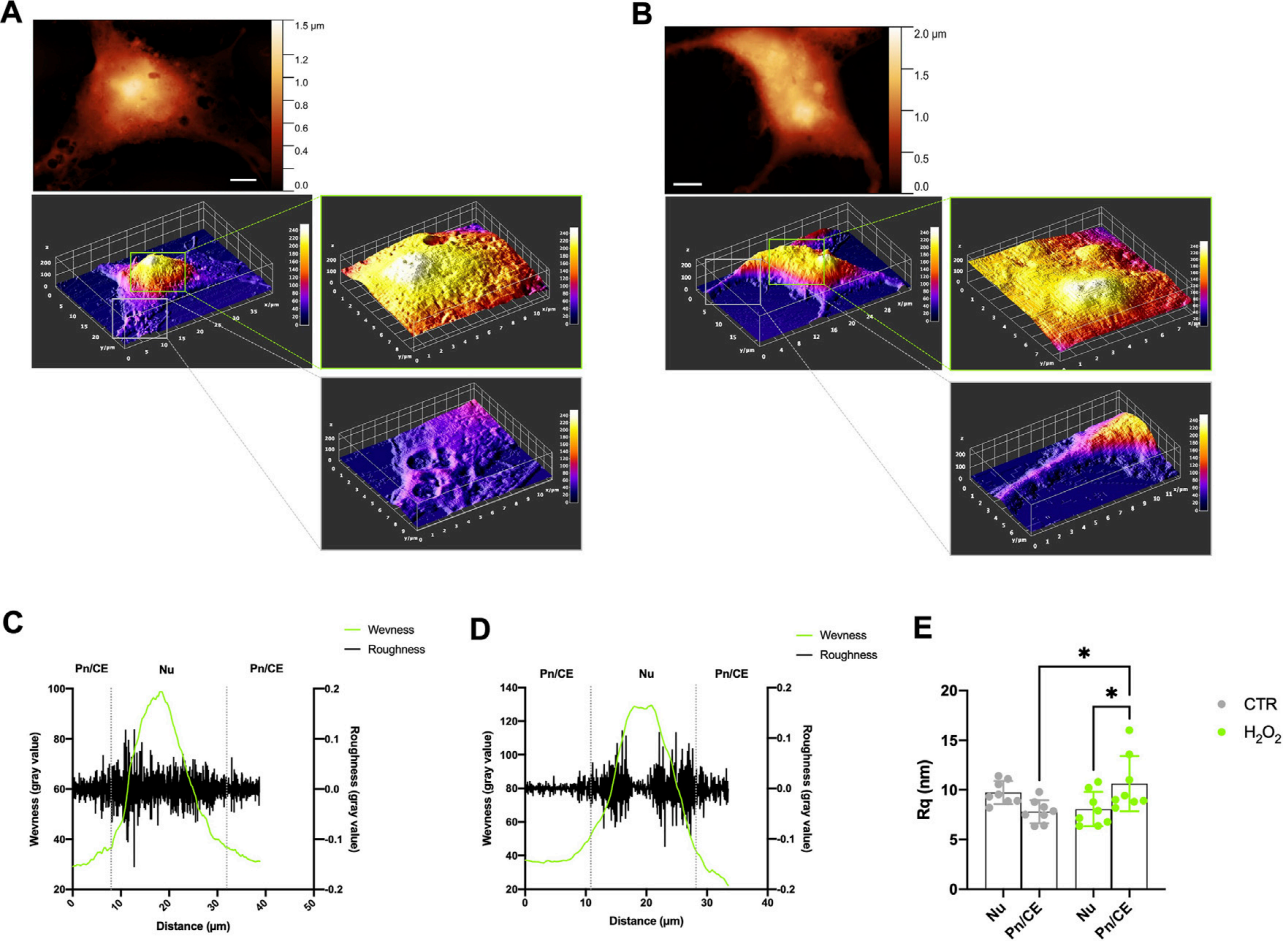
Roughness and waviness analysis performed on the AFM images. **(A)** and **(B)** example of the procedure to select the 6 × 6 microns wide regions on nuclear and perinuclear areas on one control cell (Panel A) and one H2O2 treated cell **(B)**, scale bars 5 microns. **(C)** and **(D)** example of calculation of roughness (black line) and waviness (green line) along a line onto an entire control **(C)** and H2O2 treated **(D)** cell, the grey vertical dotted lines separate the nuclear (Nu) and perinuclear (Pn/CE) regions. **(E)** roughness values obtained from all the images sampled, each dot is representative of a region (Nu or Pn/CE) for each cell, the results obtained on the control cells are shown in grey dots while the ones obtained on the H2O2 treated cells are shown in green dots. The average values of roughness obtained for the control cells are 9.8 ± 1.2 nm and 7.8 ± 1.2 nm in the nuclear and perinuclear regions, respectively. While for the H2O2 treated ones, the obtained values are 8.1 ± 1.7 nm and 10.6 ± 2.8 nm for the nuclear and perinuclear regions, respectively. The mean roughness values were statistically different between nuclear and perinuclear regions (*p* < 0.05) in the H2O2 treated samples and in the perinuclear region between control and H2O2 treated cells (*p* < 0.05).

The membrane roughness (Rq) is a key parameter that is directly related to the cellular surface arrangement and topography at the nanoscale level. It represents a direct estimation of the good attachment of the membrane to the underlying cytoskeleton and it can be used to evaluate the cellular status (Carelli-Alinovi et al., 2019) and follow the evolution of the morphological patterns by quantifying them in a reproducible way (Dinarelli et al., 2018b). In our experiments this parameter is, however, intended as a combined measure of pure membrane roughness and budding phenomena, i.e., the more extensive budding is present, the higher roughness has to be expected. In view of these considerations, we decided to evaluate the roughness in the two different areas already discussed: the nuclear (Nu) and the perinuclear/cellular edge (Pn/CE) regions of the cells. Moreover, we decided to measure the waviness on each region, to evaluate the membrane arrangement over longer lateral scales (Goic et al., 2011), then the ones used for the calculation of the local roughness. More in detail, in the case of a single cell, the waviness can be seen as a simplified profile of the entire cell. The results obtained are shown in Figure 4. In particular, in Figures 4A, B are shown the preliminary steps of the region selection and the corresponding height maps obtained for one control and one H2O2 treated cell, respectively. An example of the behavior of roughness and waviness over the entire cell is shown in Figure 4C (for one control cell) and in Figure 4D (for a H2O2 treated one). From these two examples can be clearly seen how the values of the linear roughness (black lines) are quite different between the nuclear and perinuclear regions (separated by dashed vertical gray lines). In general, the values are less scattered in the perinuclear region with respect to the nuclear one, this fact well correlates with the topography of the cell (i.e., the higher, the rougher). The roughness value, however, becomes a quantitative parameter, and thus can be used to compare different samples and regions if it is calculated over areas with the same lateral dimension and the same total number of pixels. In order to provide a quantitative analysis, we computed, for each cell, the roughness values in 2 or 3 (depending on the total dimension of the cell itself) not-overlapping 6 × 6 microns areas onto the Nu and Pn/CE regions obtaining a representative average value of roughness representative of the region. The obtained results are shown in Figure 4E. The values of roughness obtained for the control cells were 9.8+/−1.2 nm and 7.8+/−1.2 nm in the nuclear and perinuclear regions, respectively. While for the H2O2 treated ones, the obtained values are 8.1+/−1.7 and 10.6+/−2.8 for the nuclear and perinuclear regions, respectively. A first consideration of these values resides in the analysis of the statistical error obtained, that is higher in the H2O2 treated cells with respect to the control. This can be seen as a confirmation of the fact that the H2O2 exposure enhances the budding phenomena that, consequently, gives values more spreaded among the different cells. Considering only the nuclear region, there is no statistically significant difference between the untreated and treated cells average roughness values, while there is a significant increase (*p* < 0.05) in the perinuclear regions. Therefore, also the average roughness values measured on the nuclear and peripheral regions of the H2O2 treated cells are statistically different (*p* < 0.05). These two latter considerations, taken together, further confirm that the enhancement of the budding phenomena is triggered by the H2O2 exposure, but also that it is more pronounced in the peripheral region of the cell.

## Discussion

**FIGURE 5:**
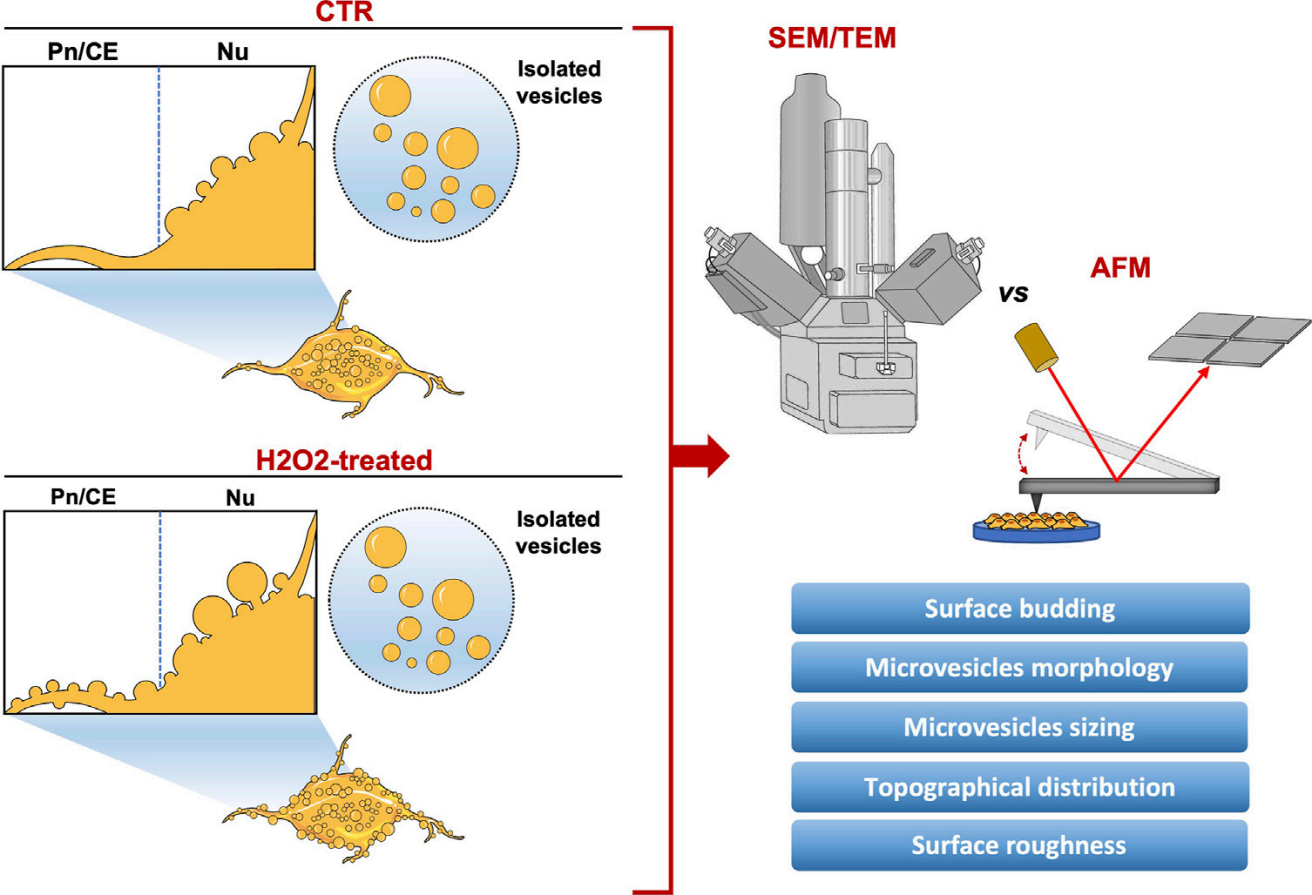
Graphical summary of the study.

In this study, we applied a reliable AFM-based method to evaluate the number, size, and distribution of budding and isolated vesicles in different cell culture conditions and treatments. To better confirm the reliability of our investigations, AFM measurements were compared with gold-standard methods globally used for the analysis of superficial budding (i.e., SEM) or ultrastructural analysis of isolated EVs (i.e., TEM). When discussing the differences between the data obtained with SEM and AFM, at least one prior consideration is necessary. From the direct comparison between the two techniques, a first evidence of the validity of the AFM system for the analysis of vesicles budding emerged. Both the methods provide perfectly comparable results, but the AFM analysis allows to highlight subtle differences in the budding process, such as the different distribution of the vesicles over the entire cell surface, probably hidden by the sample preparation procedure of the SEM. Indeed, it is evident how the cells analyzed by SEM have a much smoother surface than those analyzed by AFM, where the cell membrane appears almost completely covered in buds, particularly in the nuclear region. Perhaps, this effect is due to the different protocols necessary to obtain stable samples. The SEM preparation procedure is harder than the AFM one (Figure 5). In particular, the cell surface flattening observed in SEM micrographs is probably due to the sputter coating (a process which applies an ultra-thin coating of electrically-conducting metal in a non-conducting or poorly conducting specimen (Brodusch et al., 2018), not involved in the AFM preparation procedure. The details obtained by analyzing the samples by AFM, but not by SEM, can be crucial to fully investigate the dynamics of the budding phenomena under H2O2 exposure, compensating for the higher analysis time needed to perform the AFM images with respect to the SEM scanning (i.e., two days versus a few hours, respectively). Moreover, the AFM shows the unique capability to discriminate between the budding phenomena on different regions of the cell, thus highlighting a very peculiar effect of the H2O2 treatment that ends up increasing the budding phenomena at the cellular edges and connections. The unique capability of the AFM to measure the real height of the samples with high spatial and vertical resolutions, makes it possible to quantify the local arrangement of the membrane in a mathematical way by using the roughness analysis.

The morphological analysis of isolated vesicles is crucial to determine the quality of the isolation procedure and the presence of vesicles in the isolated fractions (Chuo et al., 2018; Hartjes et al., 2019). In this context, TEM analysis represents a gold standard method used almost universally by scientists working in the field of extracellular vesicles (Cizmar and Yuana, 2017; Malenica et al., 2021; Noble et al., 2020). However, multistep preparations required for electron microscopy can easily alter the morphology of EVs, often providing unreliable information. To overcome this problem, several new approaches for morphological analysis of vesicles have been proposed, including AFM microscopy (Sorkin et al., 2018; Skliar and Chernyshev, 2019; Bairamukov et al., 2020; Vorselen et al., 2020). An in-depth comparative study between the two methods is necessary to define the quality of the isolation procedures and evaluate the adequate morphological characterization of the new proposed technique with respect to the one considered as a golden standard. According to our analysis, the general qualitative behavior and appearance of the distributions of the fractions isolated from control and H2O2-treated cells is consistent among the two techniques, thus indicating a high degree of comparability between the two methods. However, when comparing the mean values obtained for the two distributions, the values measured by AFM are significantly higher than those obtained by TEM. This result can be attributed to two major contributions: 1) the layer of water contaminants on the surface of the specimen, due to the environmental humidity, that is always present when performing AFM measurements in air; and 2) the tip convolution, an always emerging effect when performing AFM images on small structures. Indeed, the borders of small structures are difficult to measure with high precision and, therefore, a slight overestimation of the later size (in an order of magnitude of the tip radius) is induced. In our experimental configuration, this effect can be estimated approximately as 15–20 nm. However, the values distribution is perfectly compatible among AFM and TEM data and, since the number of particles that can be counted over the same acquisition time is higher in the AFM analysis, it allows to obtain more meaningful and statistically consistent results. After all these considerations, we can conclude that the AFM can represent a valid alternative to the TEM characterization when dealing with MVs, since it is capable of delivering a higher amount of data that, however, need to be discussed in terms of absolute values. This latter consideration can be further solved by performing measurements by varying the tip radius (i.e., by performing the measurements with different tip’s radius and geometry). Indeed, this technique tends to slightly overestimate the real size of EVs but has the undoubtable advantage to avoid the exposure of the sample to an extreme vacuum environment. Moreover, this overestimation is consistent among the samples considered in this work and does not impair the relative differences observed in terms of distributions of the diameters of the vesicles: i.e., with a single, well-defined peak in the control EVs while a more spread distribution in the EVs derived from H2O2 treated cells.

Finally, we demonstrate that the budding of vesicles in control cells is quite homogeneous all over the cellular membrane, while the exposure to H2O2 had two major consequences on the budding patterns: 1) the average size of the vesicles increases both in the nuclear and in the peripheral regions, and 2) a sensible increase in the number of vesicles produced only in the peripheral area. These considerations further confirm the unique capabilities of the AFM to give local information all over the cellular membrane, from a single AFM image it is possible not only to quantify the budding of membranes on the entire cell, but also to focus on different regions, thus highlighting the localized response of the budding phenomena to the H2O2 treatment. Through the roughness analysis, we demonstrate that it is possible to quantify the local distribution of the heights through a post-processing approach that can also be automatized and gives results in perfect agreement with the ones obtained by the manual counting of the vesicles on the cellular membrane.

## Data Availability

The raw data supporting the conclusions of this article will be made available by the authors, without undue reservation.
